# Early detection of criminality concerns and the social link

**DOI:** 10.1186/s40504-014-0013-8

**Published:** 2014-05-14

**Authors:** Laurence Perbal

**Affiliations:** Centre de Recherche Interdisciplinaire en Bioéthique (CRIB), Philosophy Department, FNRS fellow & Université libre de Bruxelles, 50 av. F.D. Roosevelt, CP 175/01 1050 Brussels, Belgium

## Abstract

In modern societies, rhetoric focused on body and health is common as biomedical sciences have taken a big place in people’s lives. They must enhance the society, health and living conditions of citizens. Solving criminality problems remains a major challenge and the early detection of antisocial children - future offenders - promises to offer a solution to criminality thanks to science and medical advances. But in a democratic society that values ​​solidarism and pluralism and tends to preserve the social link, it is necessary to question the ethical relevance of this method of managing criminality. This paper proposes to analyze these questions in the light of debates that have troubled France for a number of years.

## Introduction-the debate

In 2005, the former President of the French Republic, Nicolas Sarkozy, defended a draft report on “crime prevention” which called for very early detection of behavioral disorders. He referred to a report from the *Institut national de la santé et de la recherche médicale* (Inserm), entitled “Troubles des conduites chez l’enfant et l’adolescent” or “Conduct disorder in children and adolescents” (Institut national de la santé et de la recherche médicale 2005). That report, published in September, 2005 shows that some early conduct disorders have a marked heritability, are correlated with early aggression and are predictive of future delinquency. Indeed, conduct disorder is primarily defined by the repetition and persistence of behavior patterns which flout societal rules and other people’s fundamental rights. Inserm therefore proposed that risk factors be detected at an early stage, even from early pregnancy, and to organize regular interventions in families at risk, particularly among young mothers with a first pregnancy in a precarious environment. Behavioral disorders in young children would then be recognized as early as the age of 36 months with regular medical checkups and a follow-up in the child’s health booklet.

Following Inserm’s publication, a group of citizens formed a group named Pas de 0 de conduite (1). In March 2006, it sent an open letter, accompanied by a petition, to the General Director of Inserm to denounce an “extreme medicalization of educational, psychological and social phenomena” and “confusion between social disorders and mental suffering, or hereditary disease.” This group also alerted the National Consultative Ethics Committee (Comité consultatif national d’Ethique - CCNE) and called for a critical analysis of the report.

In February 2007, the CCNE published a report on the ethical issues raised by the early detection of behavioral disorders in children (Comité consultatif national d’Ethique 2007). This brief report questions the collective expertise of INSERM and underlines three major issues:

1. On the epistemological problems and semantic ambiguity of “conduct disorder”;
2. On the neglected importance of environmental factors;
3. On the risk of stigmatization.

Can early medical screening of behavioral disorders really prevent crime? Is dealing with suffering children through formal medical diagnosis a civic and societal acceptable initiative? Or is it breaking the social link? These questions raise important and urgent ethical challenges for the citizen. Yet groups of “wise men” are those who make decisions. This debate is exemplary: it is built around two “groups of wise persons”, a group of scientific experts and the group of the Ethics Committee. Is there a real place for the popular voice in this highly elitist dynamic?

In this paper, I propose to analyze and discuss in more details the epistemological and ethical arguments rose by CCNE against the recommendations of the experts of Inserm. We will be then able to analyze the relevance of the proposal of “early detection of future offenders” regarding democratic civic values: solidarism and pluralism.

## The Inserm report

The Inserm expert group proposes to anticipate the development of major behavioral disorders linked to delinquency and crime thanks to a list of behavior-qualified problems: aggression towards people or animals; destruction of property without aggression; deceitfulness or theft; and serious violations of established rules. Thus, the idea is to identify any risk factors at an early stage: prenatal and perinatal, genetic, environmental or related to temperament and personality. This proposal is based on a meta-analysis of scientific researches showing that individuals who have conduct disorder in early childhood - as early as 3 years of age - and grow up in unstable environments combining poverty and abusive treatment have significant risks of becoming violent. Indeed, conduct disorder tends to persist with two-thirds of children thus diagnosed still being diagnosed as having the disorder in adolescence (Institut national de la santé et de la recherche médicale 2005, p.11).

The risk factors for conduct disorder are numerous and are the same for high levels of physical aggression, antisocial behavior and delinquency.

First, deleterious parental attitudes and inappropriate child-raising strategies seem to be important. “*The main factors are a parental history of antisocial behavior during adolescence, the mother being young at the birth of her first child,* (…) *family conflict, poverty, and coercive behavior towards the child by the parents*.”(Institut national de la santé et de la recherche médicale 2005, p.14; Cadoret et al. 1995; Farrington and Loeber 2000; Loeber et al. 2000; Rutter 2001).

Second, diagnosis of conduct disorder in children is commonly associated with oppositional defiant disorder (ODD) and attention-deficit/hyperactivity disorder (ADHD) in co-morbidity studies (Angold et al. 1999; Koenen et al. 2006). They are brought together under the term “disruptive behavior” in the two main international classification systems (DSM and ICD).

Third, the meta-analysis underlines the relative weight of genetic and environmental factors that specifically predispose to conduct disorder, ODD or ADHD; and conduct disorder seems to be less heritable than ADHD—about 50% (p.15; Rhee and Waldman 2002). According to the Inserm experts these findings are consistent and justify looking for vulnerability genes. In that purpose, experiments in animals and clinical research have shown that various neuromediators (serotonin, dopamine, GABA, neuropeptides, etc.) are involved in impulsiveness, aggression and violence. For example, the dopamine system is known to be involved in motor excitability and concentration (Faraone et al. 2001).

Then, according to many studies, personal variables such as temperament and personality seem key factors in the development, maintenance and severity of conduct disorder. In children, a difficult temperament (e.g. negative mood, lack of perseverance, inability to adapt to situations, readiness to be distracted, intense emotional reactions, hyperactivity and social withdrawal) is a risk factor if the child comes from a dysfunctional family (Cimbora and Mcintosh 2003, Hirshfeld-Becker et al. 2002).

Finally, the experts focus on neurocognitive deficits associated with conduct disorder in children and adolescents: impaired verbal skills and impaired executive inhibition mechanisms (Pineda et al. 2000).

From a therapeutic perspective - in addition to these predictive aspects -, the report recommends using treatment strategies which have been demonstrated to be effective, such as personal or group therapy for the child, and personal or collective parent training. Child-centered interventions are intended to promote social, cognitive and emotional skills (empathy, improve anger and impulse management, foster the ability to establish relationships with other people (especially peers) and enhance communication skills) (2005, p.40). Then if that fails, or if they are not “effective” rapidly enough, Inserm report recommends the use of medication (psychotropic stimulants and mood stabilizers) as early as 6 years of age. For example, the use of Ritalin to treat hyperactivity is indeed increasingly common.

## CCNE criticism and comments

### On the epistemological problems and semantic ambiguity of “behavioral disorder”

a. “*The ambiguity of the definition given in DSM-4* [Diagnostic and Statistical Manual of Mental Disorders] *and ICD-10* [International Statistical Classification of Diseases and Related Health Problems] *for “behavioral disorders” must be emphasized because it tends to blur the frontier between pathology and delinquency, between medical and judicial considerations*.” (Comité consultatif national d’Ethique 2007, p.3)

Indeed, conduct disorder is a psychiatric category marked by a degree of repetitive behaviors where the rights of others or social norms are violated. The disorder is often seen as the precursor to antisocial personality disorder. Symptoms include verbal or physical aggression, cruel and aggressive behavior towards other animals or people, destructive behavior, lies, fraud, vandalism and theft. Many of these terms have their origins in the judicial domain but here they are used for medical diagnostic purpose.

So, research on aggression may involve convictions for crime, fighting, violent anger, verbal aggression. It takes into account justice-related reports of convictions for violence and also, self-assessments, assessments by others (parents, peers, children, and teachers), the responses to specific questionnaires and psychiatric or clinical diagnoses (e.g. BD Hostility Inventory, the Child Behavior Checklist or the Grey and Cloninger Checklist).

Aggressive behaviors may also include the diagnosis of antisocial personality disorder and to meet this diagnosis, the individual must have some of the characteristics from the following list (DSM-5 2013):

A. Significant impairments in personality functioning manifest by:
  1. Impairments in self functioning (a or b):
    - Identity: Ego-centrism; self-esteem derived from personal gain, power, or pleasure.
    - Self-direction: Goal-setting based on personal gratification; absence of prosocial internal standards associated with failure to conform to lawful or culturally normative ethical behavior.
    2. Impairments in interpersonal functioning (a or b):
      - Empathy: Lack of concern for feelings, needs, or suffering of others; lack of remorse after hurting or mistreating another.
      - Intimacy: Incapacity for mutually intimate relationships, as exploitation is a primary means of relating to others, including by deceit and coercion; use of dominance or intimidation to control others.

B. Pathological personality traits in the following domains:
  1. Antagonism, characterized by: manipulativeness, deceitfulness, callousness, hostility;
  2. Disinhibition, characterized by: irresponsibility, impulsivity and risk taking.

Note that the same features, but at another level, are used to diagnose psychopathy and sociopathy (Longino 2001, 2013).

So, descriptions and terms are too numerous and semantic vagueness contributes to the creation of “catch-all” categories which are open doors for caricatures and stigmas. Moreover, sources based on data collected in the judicial system are certainly abundant, but they reflect evident community and social inequalities, at least in the resources invested in legal fees and therefore the probability of being convicted.

The Inserm experts write: “*In order to distinguish them from normal childhood behavior, symptoms such as physical aggression, lying and theft (which are relatively common in all small children) are only considered “pathological” if they are very frequent and persist beyond the age of 4. By adolescence, such acts tend to have more serious consequences*.” (2005 p.37) Indeed, conduct disorder may be expressed in the form of acts of delinquency which end with the adolescent appearing before the courts. So, boundaries between pathology and delinquency are clearly blurred on various levels. This criticism is generally applicable for the entire science of human aggressive behavior and the Inserm expert report underdeveloped these critical aspects.

However, to compensate for the lack of clarity and the proliferation of terms, more and more studies use “endophenotypes” that is to say; they are trying to “break up” the various forms of antisocial behaviors into stable phenotypes in order to refine the diagnosis. Gottesman and Gould write in 2003: “*the phenotypic output from the brain,* i.e.*, behavior, is not simply a sum of all its parts. It stands to reason that more optimally reduced measures of neuropsychiatric functioning should be more useful than behavioral “macros” in studies pursuing the biological and genetic components of psychiatric disorders*.” For example, a number of children with early conduct disorder seem not react emotionally. They have high scores of callous and unemotional traits (CU), which are elements of the diagnosis of psychopathy in adults. They lack empathy and guilt feeling, and they seem usually indifferent to punishment. 30% of early antisocial children have CU traits. The amygdala and several other regions of the prefrontal cortex show diminished activity (Frick and White 2008). The heritability of antisocial behavior in children with CU traits is higher (0.67) than children not displaying these kinds of traits (0.3) (Viding *et al*. 2005).

b. “*Another epistemological problem is connected to the very different nature of behaviors considered to be symptoms and diagnostic criteria of “behavioral disorders”. Can one — without giving the matter a second thought — consider as given and obvious that the tantrums of a child of three or four are early symptoms predicting a linear progression, ten or fifteen years later, to violent conduct (rape, armed robbery,* etc.*) arising from the same biological causality?*” (Comité consultatif national d’Ethique 2007, p.3)

The Inserm report did not take into account this difficulty. According to them, “*the question of whether these two forms* [of behavioral disorders] *are fundamentally different or whether they merely represent differential expression of the same underlying vulnerability remains unresolved.*” (Institut national de la santé et de la recherche médicale 2005, p.36) However, the violence of a child is not that same as that of an adult, neither in its biological and environmental causes nor in its effects. In fact, many studies highlight the etiological diversity of development patterns of aggression. For example, if antisocial behaviors developed during adolescence are primarily influenced by the environment, and in particular the non-familial environment, the same behaviors exhibited in childhood and beyond adolescence seem more influenced by genes (Rhee and Waldman 2002, Perbal 2011).

Finally, “*confusion between causality and correlation is an ever present temptation and a pitfall for any kind of research*” (Comité consultatif national d’Ethique 2007, p.4).

Indeed, the predictive perspective is based on the confusion between correlation and causation. It is important to differentiate “risk factor” - which is a statistical tool, a correlation - and “causal relationship” - which reflects a determination link. A positive correlation may suggest a causal relationship but it is not necessarily the case. The scientific argument must not caricature itself by abusing statistical tools. Thus, the existence of some risk factors - e.g. precarious family situation or early behavioral disorder - increases the probability for a young child to develop a conduct disorder; this has been shown by statistical studies on large populations. However, this mathematical result is not informative at the individual level - the only relevant scale from a preventive point of view – because correlation between abusive environment and conduct disorder may not be realized for a given child. The existence of a correlation underlines a link that is not deterministic but probabilistic, since its predictive value at the individual and preventive level is limited. So the screening procedure for individual preventive purpose is at the same time meaningless and useless.

Thus, the conclusion of CCNE group is clear: early detection of antisociality in a predictive sense is not relevant and could even be dangerous for society. Moreover, in accordance with the epistemological problems and due to the current state of knowledge, it is not possible to draw objective conclusions on the biological origins of aggressive and antisocial behaviors that go beyond social and ideological prejudices.

In my point of view, even if epistemological problems cannot be ignored, there are anyway very interesting researches that investigate biological markers of antisociality.

#### Antisociality and interactive determinism

On that topic, Carey and Gottesman write: “*We accept as a given that there is a noteworthy genetic influence on ASB no matter how it is defined. In terms of behavioral research, the magnitude of that genetic influence is substantial, but so is the impact of environment, broadly defined to include pre- and post-natal, physical (*e.g.*, anoxia, fetal, alcohol syndrome, or crack) as well as psychosocial (*e.g.*, quality of parenting, ethnic culture, or religion elements*” (2006).

For example, since the 1990s, researchers have been interested in a locus that produces a variant of the enzyme called MAO of the brain (monoamine oxidase): MAOA (monoamine oxidase A) (Brunner *et al*. 1993). Its function is to control the amount of neurotransmitters, including serotonin. The correct transmission of messages between nerve cells and between nerve cells and muscles is disrupted if neurotransmitters are too numerous. A number of studies have shown a statistical (positive correlation) or causal (knock-out experiments in mice) link between low levels of MAOA and aggressive behavior, mental retardation, behavioral lack of self-control or addiction (Cases *et al*. 1995, Shih and Thompson 1999).

Moreover, Andreas Meyer-Lindenberg showed in 2006 that amygdales in individuals showing a low MAOA activity have a higher activity. When they observe pictures of angry or frightened faces, and when they reminisce about negative memories, low active gene carriers show high levels of amygdale activity and lower activity in the prefrontal cortex that regulates the amygdales responses to stimuli. “*Genes are major contributors to many psychiatric diseases, but their mechanisms of action have long seemed elusive. The intermediate phenotype* [or endophenotype] *concept represents a strategy for characterizing the neural systems affected by risk gene variants to elucidate quantitative, mechanistic aspects of brain function implicated in psychiatric disease*.” (Meyer-Lindenberg and Weinberger 2006; Meyer-Lindenberg et al. 2006) Some brain imaging research projects have also revealed that the hippocampus is both structurally and functionally different. Memory problems may then be associated with aggressive behavior because they are both markers of dysfunction of the hippocampus (Raine *et al*. 1997).

Then, a growing literature in neuropsychology suggests that individuals who commit antisocial and violent acts may have neuropsychological trauma. For example, some research shows that early visual-spatial problems (in the right hemisphere) may predispose an individual to permanent antisocial behavior due to interference with emotional recognition and regulation. Poor recognition by the child of facial expressions can limit the expressive responses and disrupt the reciprocal relationship between mother and child. Disruption of this first human relationship may predispose the child to reduced emotional sensitivity (Young *et al.*^2002^). Similarly, a lack of maternal expressed emotion toward the child is associated with antisociality and poor recognition of negative affect processes and signals (such as fear and anger) may contribute to an escalation of aggressive responses (Caspi *et al*. 2004).

Finally, studying interactions between genes and the environment is essential. For example, in August 2002, Science published a major study showing that the association between low active allele of MAOA gene and behavioral disorders, a relative high rate of conviction and antisocial and violent behaviors is highlighted when the gene carrier has itself been a victim of abuse and violence in childhood. No significant correlation was observed among individuals abused in childhood and producing many MAOA or non-abused individuals producing MAOA in small quantities. It seems that the increase in MAOA activity reduces the probability that an individual, who experienced violence in childhood, develop a violent personality. These two interacting factors affect the feelings and emotional reactions of children (Caspi *et al*., 2002). Similarly, in adoptive family with problems (divorce, separation, psychiatric diseases…), adopted children at risk - that is to say having biological parents exhibiting conduct disorders - seem more likely to develop antisocial behaviors (Cadoret *et al*. 1995, Button *et al*. 2005).

In summary, an antisocial act or conduct disorder is a contingent event. This is a behavior that is the result of the interaction of an individual with his environment and with others. Some genetic markers (MAOA) and environmental factors (violence in childhood, mothering effect) are correlated to the development of a predisposition to antisocial feelings and acts. The expression of this predisposition seems facilitated by the interaction of various risk factors. Studies of the effects of factor interactions on behavior variance are a starting point for developmental approaches. In this area, the neurosciences have made some interesting discoveries. Disturbances in brain areas associated with emotion (orbitofrontal cortex, anterior cingulate cortex, amygdala) may be involved in the emergence of aggressive behavior. Disorders related to the recognition of facial emotions might facilitate antisociality due to reduced sensitivity to the emotions of others. In addition, there is no single form of antisociality and the developmental origins of each form are not uniform.

I think that these kind of studies are promising because they really respect the fact that the causes of aggression are multiple, contextual and interactive (Tabery 2009). “*The new model views genes and the environment as engaging in a life-long dance. The type of dance, its movements, and its tempo change, sometimes very quickly. The lead, moreover, varies from moment to moment. We call it the “tango” model.”* (Carey and Gottesman 2006) I call it the “interactive determinism” (Perbal 2010).

### On the neglected importance of environmental factors

Many of these studies are summarized in the INSERM report. But for the CCNE, the place of the environment in the genesis of antisocial behavior has not been sufficiently taken into account: “*The expert report’s tendency to find genetic causes or cerebral susceptibilities of a neurobiological nature playing a preponderant role in future development to violent forms of delinquency, would seem to postulate and beg the question rather than take full account of the available data. Social or environmental factors would seem to be at least as decisive a factor for ulterior behavior as the individual genetic, neurobiological or psychological factors affecting a particular child*.” (2007 p.4) According to the CCNE, this excessive focus on biology can probably be explained by the choice of a group of experts mainly from the domain of biology and psychiatry. This group therefore did not satisfy the interdisciplinary requirement required by the multiple issues and dimensions involved in this topic, which is at the same time scientific, medical, human, psychological and sociological.

If I do not agree with them on the fact that Inserm report ignores significant research on relationship between genes and the environment, I cannot deny that they develop an eminently medical approach in the analysis of behavior disorders in young children. Their role is to detect, diagnose and treat “psychiatric” disorders. In that context, any deviation to standard social behaviors tends to be equated with symptoms of mental illness in the making. “*Truancy, rudeness at school and academic failure are all associated with conduct disorder. The group of experts recommends raising the awareness of teachers about the various behavioral manifestations of conduct disorder, and encouraging them to collaborate with professional health care providers on early intervention in children and adolescents*.” (Institut national de la santé et de la recherche médicale 2005, p.37)

It is essential to not medicalize excessively misbehaving - in both diagnostic and therapeutic approaches - in relation to a certain standard of discipline. For some turbulent child, testing the limits or denying the social norm may also be part of the construction of their identity. This would be a dangerous drift of the modern tendency towards the naturalization of bodies and minds.

Indeed, speeches focusing on the body and on individuals’ health are increasing. Naturalization of the human being is everywhere and behavioral problems become objects of analysis in the medical field. The importance of medicine in our lives is real and the consequences are barely imaginable in the future. In particular, it should avoid taking the easy route of giving psychotropic or anxiolytic drugs to young children. Excessively early medication could hide disorders and prove useless in curing the background malaise by merely hiding the effect. In addition, these treatments are risky for the cognitive, neuropsychological and emotional development of a child whose brain is growing. The risk of dependence is not trivial either. Cognitive behavior therapy (CBT) might be a good alternative as individual treatment. CBTs are “active therapies” because psychotherapists do not only listen to the patient, but share information with him, offers therapeutic techniques, tips, etc. These techniques include imagined exposure (virtual reality therapy or in vivo therapy), relaxation or cognitive reframing. The latter often targets the working memory which is an essential capacity for daily cognitive activities requiring reasoning, comprehension and learning. A deficit of working memory is found in many disorders including learning disabilities, attention disorders and hyperactivity disorder (ADHD). In recent years, computer software – called COGMED - offers cognitive remediation games to patients. It seems to be an effective therapeutic tool (Beck *et al*. 2010).

### On the risk of stigmatization

The French Inserm experts write: “*The group of experts recommends using health visits with their systematic pediatric monitoring from young childhood through adolescence to improve the efficiency of screening of both conduct disorder and its risk factors and simultaneously correct any previous incorrect labeling of normal behavior as pathological. The group of experts recommends introducing certain age-appropriate items into the parameters recorded in a child’s Health Records in order to identify signs that may indicate nascent conduct disorder. These items might cover symptoms, such as physical aggression (fighting, attacking, hitting, biting or kicking), oppositional behavior (disobedience, lack of remorse or failure to modify conduct), and hyperactivity (inability to stay put or wait for his turn, constant fidgeting)*.” (2005, p.39) The report recognizes that these observations are in themselves insufficient for a formal diagnosis of conduct disorder.

Anyway, and even if early detection with inscription in a health booklet may have some benefits – for preventive and therapeutic care – it is not able to counteract the risk of negative effects for children in finding themselves stigmatized so early. In any event, that stigmatization, instead of serving their interest by promoting greater social and educational support, could strengthen discomfort.

Labeling is very likely to maintain the confusion between prevention and stigma. The conceptual leap - from “children at risk” to “dangerous children” - is a pernicious security drift. CCNE writes: “*There are circumstances — and therein lies the problem with “behavioral disorders” — where the medical may be tempted to step into the shoes of the judiciary and decide to devote their time to preventing delinquency, forgetting that their primary mission is to alleviate suffering*.” (2007 p.5)

Moreover, antisocial behavior does not necessarily lead to later criminal or psychopathic behavior and many crimes are rather the result of a combination of social factors.

In these circumstances, breaking down medical confidentiality cannot be justified, especially when the purpose is to communicate these biological and medical data to governmental officials. Such information in health booklets encourages discrimination that is based on criteria with no certain association with future antisocial behaviors. Preventive medicine should not extend into the judiciary.

### Preserving the social link

The security-related proposal of early detection of criminality meets a pressing social need. Nicolas Sarkozy said in 2005: “*We must act sooner, detecting violence issues at a younger age. In kindergarten, in primary school, there must be teams to handle these problems*.” (*Le Parisien,* December 2, 2005) Of course we must ensure the safety of citizens but do antisocial young children have to be targeted? Is early detection of future offenders the mark of an acceptable civic involvement or a rupture of the social link?

First, besides the abusive medicalization of behaviors, one of the major difficulties in the early diagnosis of antisocial personality is obviously the negative consequences that a negative outlook can have on the cognitive and affective abilities of a child. For example, individuals with low self-esteem in childhood and adolescence may avoid social relationships with peers, or friendships and sentimental relationships. In addition, it has been shown that children and adolescents with low self-esteem look for different forms of antisocial behavior as an escape from their discomfort. This isolation reinforces the feeling of being unwell. Self-esteem is then an important factor associated with conduct disorders. Adolescents with low self-esteem seem more likely to be convicted for crime in adulthood, to develop major depression, anxiety, addiction to tobacco, premature departure from school and a long period of unemployment (Trzesniewski *et al*. 2006). Even if this kind of research is plagued by the epistemological problems reported earlier, it seems clear that labeling may endanger self-esteem and then, doing worse than expected.

Moreover, the labeling of children would be a perverse slip from preventive medicine to predictive medicine and to judicial; turning a child victim into a guilty party. “Correlation” does not equate to “causation” and to prevent is not to predict. Preventive medicine that would take care early and appropriately of suffering children should not be confused with predictive medicine that seems to predetermine - here in a negative way - the fate of children. Children at risk must be supported in the present rather than being turned from crime in the future because suffering is real while the offending future of troubled children is hypothetical.

Thus, the early detection of future offenders is an approach that is scientifically and epistemologically questionable. Moreover, it cannot be regarded as an expression of honorable civic commitment. Stigmatizing a group of people with specific (genetic or behavioral) traits puts civic solidarity in danger. The social bond is broken when the conceptual leap between prediction and prevention, between caring for a person in danger and stigma of a potentially dangerous person is made. Thus, we share many of the critical positions of the CCNE. Certainly, wanting to identify children in need quickly is an honorable approach, at least if the goal is to improve the living conditions of these children and to understand their behavioral disorders. However, social support should not involve the labeling of children because speeches suggesting the relevance of early detection of antisociality tend to break social ties and solidarism. In general, it is often the case with discourse on body and health because we are no longer defined by our sociality and our “human nature” is likewise naturalized.

## Conclusions

Registered in the draft law on the prevention of crime, the principle of early detection of conduct disorders that may lead to delinquency in adolescence was removed in June 2006. In December 2006, Inserm was committed to submit its collective expertise to ethics and scientific committees.

However, is the proposal for the early detection of antisocial children an abusive naturalization of a strictly social phenomenon? No, there are some interesting studies in favor of interactive determinism of antisociality. But on the one hand, forms of antisociality are multiple and on the other hand, the effects of early detection may have more negative effects than positive. There is a great risk of stigmatizing a turbulent child through abusive medicalization. Testing the limits or denying the social norm may also be part of the construction of a social and emotional child. Thus, from a utilitarian perspective, the risks (for children to be stigmatized and for the construction of their identity, for the use of such data, for endangering pluralistic values and solidarism) are too numerous compared to the expected benefits (screening for would-be criminals, treatment of children who are suffering; educational caring). Crime prevention must be built on more solidarity and substantive action rather than a “predictive labeling”.

Society must develop health care and psychological counseling for parents in difficulties, including disadvantaged single mothers (teenage pregnancy, addiction situation, past violence etc.). The famous biologist Lionel Penrose already said in the early 20^th^ century that the value of a society depends on how it takes care of its weakest citizens (Penrose 1934). It must also develop greater coordination of medical and social professionals, from pregnancy and after birth. Parents, pediatricians, psychologists, psychiatrists, teachers, counselors, educators, physicians and school nurses should be involved in the design and monitoring of such measures. Cognitive games do exist to treat hyperactivity or to stimulate brain regions linked to emotions; it is therefore important to inform agents of education on these tools of cognitive restructuring. In addition, with regard to children themselves, existing care structures (preventive nurseries, therapeutic gardens, adolescent house) must be supported. For example, preventive nurseries take care of children living in economically, culturally or emotionally precarious environments. It is an ideal place for children who need special monitoring, which have psychiatric disorders, or behavioral disabilities. It is open 24 hours a day, 7 days a week and even offers to fragile families educational counseling at home. This kind of initiative should be encouraged.

Let me conclude with a last remark. This is an important and urgent ethical challenge for the citizen but yet, groups of “wise men” are those who make decisions. This debate on early screening of antisocial disorders is exemplary: it was built around two “groups of wise persons”, a group of scientific experts and the group of opponents of the Ethics Committee. Is this the mark of elitist ethics indifferent to the popular vote or does the latter have a place in the debate? It seems to me that the engaged citizen can make his or her voice heard. In fact, the controversy surrounding the early detection of antisociality is quite promising example for the proper functioning of the system. The story was this: a group of scientists issued a report and recommendations on the path to be followed by the political system; subsequently, citizens disapproved, reacted, sounded the alarm, mobilized and invited the national Ethics Committee to look into the question; the ethics committee did its work, analyzed and produced an opinion. These reactions have led the scientific institution to review its operational and ethical framework; and provided to politicians another look at the original scientific report. I find most ethical agents have been involved in this scenario: scientists, citizens, ethicists and politicians. On the form and management discussion, I think the system has worked quite well.

On the nature of the issue, we must nevertheless remain alert and cautious, especially because the breakdown of social ties through the medicalization and technification of the body often occurs gradually. For example, the status of disabled people has changed in Europe since the 1970s and the widespread development of abortion techniques. The law has changed and attitudes have gradually done the same: abortion seems the decision to take in case of disability (in the interests of the quality of life of the unborn child and parents). The change of attitude regarding disability here is at some points a consequence of progressive developments of science and technology (Julian-Reunier and Bourret 2006). The situation is however not as simple as calculating the cost/benefit ratio and there are associations that try to provide an alternative to parents who wish to keep the fetus but need to be relieved and reassured. This kind of initiative must also be encouraged by a society that values solidarity. To maintain social ties, solidarism and pluralism must be preserved; they are two characteristics of modern democratic society. They cannot be endangered by the technification of human beings and its tendency of putting aside sociality in the definition of what we are. Proper ethical practices must be thought of from a collective and civic perspective. Human beings are becoming the subject of science and technology as well as their author; anyway, they remain capable of ethical and moral choices consistent with the democratic civic values that they have chosen.
